# The role of mmu‐miR‐155‐5p‐NF‐*κ*B signaling in the education of bone marrow‐derived mesenchymal stem cells by gastric cancer cells

**DOI:** 10.1002/cam4.1355

**Published:** 2018-02-14

**Authors:** Mei Wang, Fang Yang, Rong Qiu, Mengchu Zhu, Huiling Zhang, Wenrong Xu, Bo Shen, Wei Zhu

**Affiliations:** ^1^ Key Laboratory of Medical Science and Laboratory Medicine of Jiangsu Province School of Medicine Jiangsu University 301 Xuefu Road Zhenjiang Jiangsu China; ^2^ Department of Laboratory Diagnostics Hebei Medical University 361 Zhongshan Road Shijiazhuang Hebei China; ^3^ Department of Oncology Jiangsu Cancer Hospital Affiliated to Nanjing Medical University Nanjing Jiangsu China

**Keywords:** Bone marrow‐derived mesenchymal stem cells, Gastric cancer, miR‐155‐5p, NF‐*κ*B p65, tumor microenvironment

## Abstract

Bone marrow‐derived mesenchymal stem cells (BM‐MSCs) are important precursors of tumor stromal cells. Previously, we have demonstrated that miR‐155‐5p inhibition directly induced transition of BM‐MSCs into gastric cancer‐associated MSCs. Whether miR‐155‐5p is involved in the education of BM‐MSCs by gastric cancer cells has not been established. Murine BM‐MSCs (mMSCs) were isolated and grown in conditioned medium derived from gastric cancer cell line MFC (MFC‐CM). The tumor‐promoting phenotype and function of mMSCs were detected by immunofluorescence staining, quantitative reverse transcription‐polymerase chain reaction (*q*RT‐PCR), cell colony formation assay, transwell migration, and invasion assays. Luciferase reporter assays and western blot analyses were conducted to reveal the relationship between nuclear factor kappa‐light‐chain‐enhancer of activated B cells (NF‐*κ*B) p65 and mmu‐miR‐155‐5p. miRNA mimics, inhibitor, and the NF‐*κ*B inhibitor pyrrolidine dithiocarbamic acid (PDTC) were used to evaluate the role of miR‐155‐5p‐NF‐*κ*B signaling in the education of mMSCs by MFC‐CM. We successfully established the education model of mMSCs by MFC‐CM and found that mmu‐miR‐155‐5p expression levels were reduced in mMSCs. Mimicking this deregulation by transfecting miRNA inhibitor into mMSCs produced a similar effect as that of MFC‐CM on mMSCs. NF‐*κ*B p65 was validated as a target of mmu‐miR‐155‐5p, which also negatively regulated NF‐*κ*B activation. Inhibition of NF‐*κ*B activation by PDTC abolished the effect of the miRNA inhibitor on mMSCs. mmu‐miR‐155‐5p overexpression partially blocked the effect of MFC‐CM in educating mMSCs, while PDTC treatment completely eliminated MFC‐CM activity. These results indicate that miR‐155‐5p is not the sole miRNA mediating the education of BM‐MSCs by gastric cancer cells, but downstream NF‐*κ*B signaling is indispensable for this process.

## Introduction

Mesenchymal stem cells derived from bone marrow (BM‐MSCs) exhibit the properties of selfrenewal, multidifferentiation potential, immunomodulation, and marked tropism for inflammatory sites 1, 2, 3. Over the past decades, BM‐MSCs have been adopted as potential therapeutic vehicles for gene therapy in various diseases, including cancer 4. Given research developments in the field of tumor microenvironment, researchers have recognized that tumor development is driven not only by changes in cancer cells themselves but also by contributions of cancer‐associated stroma 5. Increasing evidence has gradually revealed that BM‐MSCs can be mobilized and integrated into the tumor stroma to promote tumor progression 6, 7, 8.

The tumor microenvironment encompasses a variety of stromal cells, such as cancer‐associated fibroblasts (CAFs), endothelial cells, MSCs, immune cells, and so on. Among these stromal cells, CAFs are the most prominent cell type and their origin has gained increasing attention in recent years 9. Accumulating studies have demonstrated that BM‐MSCs can be incorporated into primary tumors and differentiated into CAFs in vivo 8, 10, 11. In vitro*,* it is widely accepted that cancer cell‐conditioned medium is an important cue for differentiation of BM‐MSCs into CAF‐like cells 12, 13, 14, 15. Recently, cancer tissue‐derived MSCs have been successively isolated and characterized in many solid tumors, including gastric cancer 16, ovarian carcinoma 17, hepatocellular carcinoma 18, and pancreatic cancer 19. Cancer cells also significantly induce BM‐MSCs to acquire cancer tissue‐derived MSC‐like phenotype and function in a paracrine manner in vitro 20, 21.

A genome‐wild analysis of breast cancer stroma suggests that epigenetic modifications frequently occur in tumor stromal cells 22. microRNAs (miRNAs) are important epigenetic regulators which have been demonstrated to be involved in reprogramming normal fibroblasts (NFs) into CAFs. For instance, Mitra et al. 23 reported that modifying the expression of deregulated miRNAs including miR‐241, miR‐31, and miR‐155 reprogrammed NFs into CAFs in ovarian cancer. Shen et al. 24 found that upregulation of miR‐1 and miR‐206 and downregulation of miR‐31 induced a functional conversion of NFs into CAFs in lung cancer. Pancreatic cancer cells secreted microvesicles containing miR‐155‐5p to reprogram NFs into CAF 25. However, the study of miRNAs involved in regulating the transition of BM‐MSCs into tumor stromal cells is still in its infancy.

Gastric cancer tissue‐derived MSCs (GC‐MSCs) were first isolated and characterized by our research group 16. Our previous work showed that miR‐155‐5p levels were aberrantly downregulated in GC‐MSCs compared to BM‐MSCs. Knockdown of miR‐155‐5p by miRNA inhibitor triggered the transition of BM‐MSCs into GC‐MSC‐like cells 26. However, whether miR‐155‐5p is conserved and serves as a key miRNA mediating the education of BM‐MSCs by gastric cancer cells is not well established.

In this study, we isolated murine bone marrow‐derived MSCs from 615 mice (mMSCs) and treated them with conditioned medium from the mouse gastric cancer cell line MFC (MFC‐CM) to establish the in vitro education model. We then determined the change in mmu‐miR‐155‐5p expression levels in mMSCs and evaluated the role and mechanism of mmu‐miR‐155‐5p in the education of mMSCs by gastric cancer cells.

## Materials and Methods

### Cell culture

Murine bone marrow‐derived mesenchymal stem cells (mMSCs) from 615 mice were isolated and characterized as previously 27. Briefly, bone marrow cells from the six‐week‐old 615 mice were collected and cultured in Dulbecco's modified Eagle's medium (DMEM) containing 15% fetal bovine serum (FBS) (Gibco, Grand Island, NY, USA) at 37°C in humid air with 5% CO_2_. When the confluence of cells reached 80%, adherent cells were trypsinized and replanted in new flasks for further expansion. The MSCs at the third passage were used for surface markers detection and multilineage differentiation capacity analysis. All the procedures were approved by the Institutional Animal Care Committee of Jiangsu University. Gastric cancer cell line MFC from 615 mice was purchased from Cell Bank, Chinese Academy of Sciences (Shanghai, China) and cultured in DMEM supplemented with 10% FBS at 37°C in humid air with 5% CO_2_.

### Cells conditioned medium preparation

For gastric cancer cells, 5 × 10^5^ of MFCs were planted in 10 cm dish (Corning, NY, USA) for 48 h. When the confluence of cells amounted to 70%, cell culture medium was harvested.

For mMSCs, 5 × 10^4^ of mMSCs were seeded in each well of six‐well‐plate (Corning) and attached overnight. After treated with MFC‐CM or transfected with oligonucleotides for 48 h or pretreated with pyrrolidine dithiocarbamic acid (PDTC, Sigma‐Aldrich Shanghai Trading Co. Ltd, Shanghai, China) for 2 h before treated with MFC‐CM or transfection, cell culture medium was removed and refreshed for 24 h and then the cell culture supernatant was collected. All of the medium were then centrifuged at 1500 rpm for 10 min and filtered through a 0.22‐μm membrane (Merck Millipore, Darmstadt, Germany) and stored in −80°C until use.

### Immunofluorescence staining

mMSCs were fixed by 4% paraformaldehyde for 15 min and washed three times, then incubated with the primary antibodies against alpha‐smooth muscle actin (*α*‐SMA) (Boster Biological Technology Co. Ltd, Wuhan, China) and fibroblast activation protein (FAP) (Abcam, Cambridge, MA, USA) at 4°C overnight and followed by Cy3‐conjugated antirabbit secondary antibodies (Invitrogen, Carlsbad, CA, USA). Finally, Hoechst33342 was used for nuclear staining. The images were acquired with a microscope (Olympus, Tokyo, Japan).

### RNA extraction and quantitative RT‐PCR (*q*RT‐PCR) analysis

Total RNA was extracted by miRNeasy Mini Kit (QIAGEN, Hamburg, Germany). *q*RT‐PCR of mmu‐miR‐155‐5p and several cytokines mRNAs were performed using miScript II RT Kit and miScript SYBR Green PCR Kit (QIAGEN) according to the manufacture's instruction. The relative expression levels of miRNAs and mRNAs were normalized to the expression of RNU6B and *β*‐actin, respectively. RNU6B and miRNA primers were purchased from QIAGEN. The amplification fluorescence signals were detected by the Bio‐Rad fluorescence thermal cycle. mRNAs primers sequences and *q*RT‐PCR conditions are listed in Table S1.

### Cell transfection

Oligonucleotides including mmu‐miR‐155‐5p inhibitor (inhibitor), inhibitor negative control (INC), mmu‐miR‐155‐5p mimics (mimics), and mimics negative control (MNC) were synthesized and purified by Genepharma (Shanghai, China). Transfection was conducted using Lipofectamine 2000 (Invitrogen), and the concentration of inhibitor and mimics were 100 nmol/L and 5 nmol/L, respectively. Sequences of these oligonucleotides are listed in Table S2.

### Plate clone formation assay

MFCs (1.5 × 10^3^) were seeded in each well of a six‐well plate and attached overnight. The culture medium was replaced with mMSCs‐CM and changed in every 3 days for 10 days. The cell colonies were stained with crystal violet and photographed. The colony containing the number of cells more than 50 were counted.

### Transwell migration and invasion analysis

MFCs (1 × 10^5^) were planted in each well of a six‐well plate and cultured in mMSCs‐CM for 48 h. Then, these treated MFCs were collected. MFCs suspended in serum‐free medium were planted into the top chambers (8‐μm pore size, Corning) with 10% FBS in DMEM in the bottom chamber as a chemoattractant. For the migration assay, 6.0 × 10^4^ of MFCs were planted into the top chambers and incubated for 10 h. For the invasion assay, 1.5 × 10^5^ of MFCs were planted into the top chambers precoated with matrigel (BD Bioscience, San Jose, CA, USA) and incubated for 12 h. The migrated or invasive cells were photographed and counted under a microscope at least six fields.

### Luciferase reporter assay

Positions 171–178 of 3ʹ untranslated coding regions (UTR) in the murine nuclear factor kappa‐light‐chain‐enhancer of activated B cells (NF‐*κ*B) p65 mRNA were predicted as mmu‐miR‐155‐5p target sequences by TargetScan algorithm (http://www.targetscan.org). Fragment of NF‐*κ*B p65 mRNA 3′UTR containing the binding sites or mutant target sites was synthesized and purified by Invitrogen. Sequences of these oligonucleotides are listed in Table S2. These oligonucleotides were annealed and constructed into pmirGLO dual‐luciferase miRNA target expression vector (Promega, Madison, WI, USA) (luc‐NF‐*κ*B p65 3′UTR). NF‐*κ*B luciferase reporter plasmid was purchased from Shanghai Yi Sheng Biotechnology Co. Ltd. MFCs plated in 24‐well plates were cotransfected with mmu‐miR‐155‐5p mimics, inhibitor or their corresponding NCs, and reporter plasmids using Lipofectamine 2000. The luciferase activities were quantified 48 h after transfection using Dual Glo Luciferase Assay System (Promega) and normalized to that of Renilla luciferase (Promega).

### Western blot analysis

Protein lysates of cells were separated by 12% SDS‐poly acrylamide gel electrophoresis (SDS‐PAGE) and transferred to 0.45 μm PVDF membranes (Merck Millipore), which was further blocked with 5% bovine serum albumin (BSA) and incubated with the primary antibodies against phospho‐NF‐*κ*B p65 (p‐NF‐*κ*B p65) and NF‐*κ*B p65 (Cell Signaling Technology, Inc., Danvers, MA, USA) as well as the secondary antibodies (Bioworld Technology, Inc., Nanjing, China). The protein band signal was visualized using HRP substrate (Merck Millipore) and analyzed using MD Image Quant Software. GAPDH was used as the loading control.

### Statistical analysis

All experiments were conducted at least in triplicate. Data were presented as means ± SD. Student's *t*‐test, one‐way analysis of variance (ANOVA), and post hoc testing (Tukey test) were performed using Graph‐Pad Prism 5 software to analyze the data. *P* value less than 0.05 was considered to be significant.

## Results

### Characterization of bone marrow‐derived mMSCs from 615 mice

**Figure 1:**
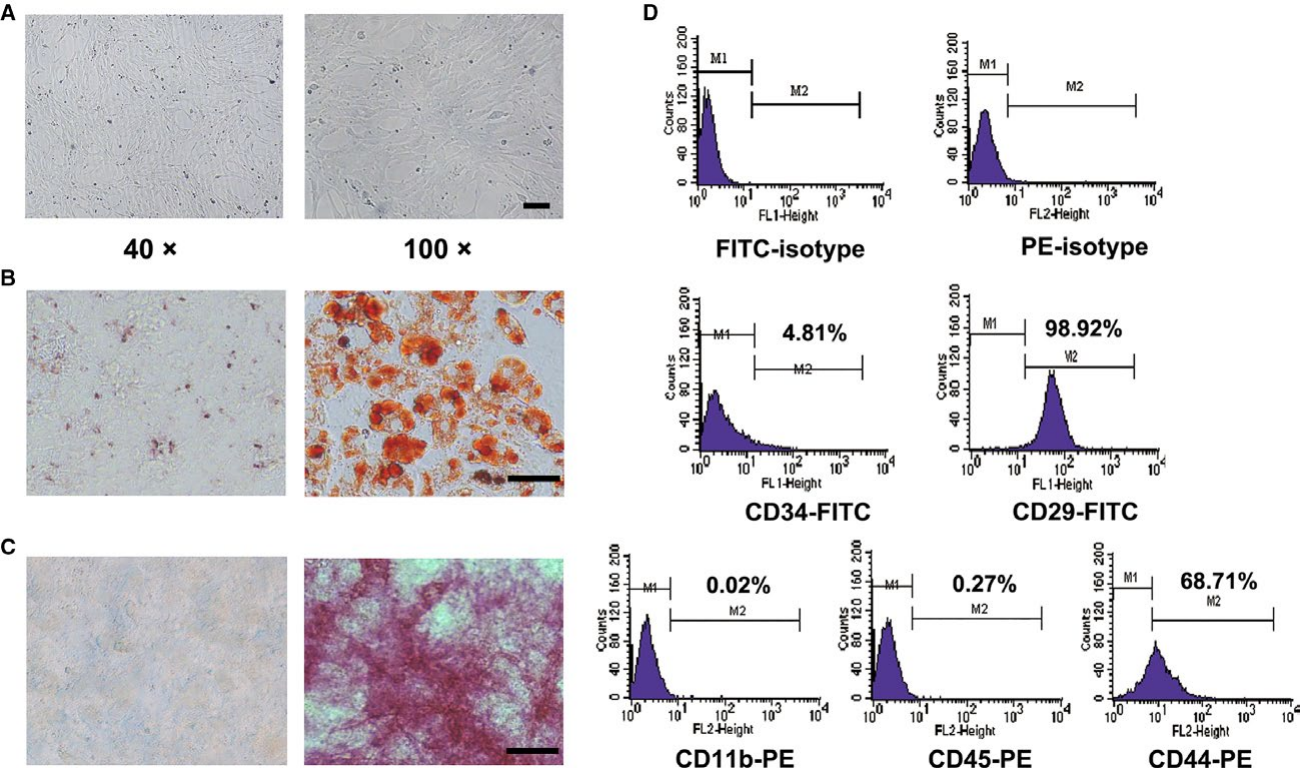
Morphology, phenotype, and multidifferentiation potentials of mMSCs. (A) Morphological appearance of murine bone marrow mesenchymal stem cells from 615 mice (mMSCs) at the third passage. Magnification: Left, ×40; Right, ×100, Scale bar = 100 μm. (B) Oil Red O staining detection for adipogenic differentiation. (C) Alkaline phosphatase staining detection for osteogenic differentiation. Magnification: ×200, Scale bar = 50 μm. (D) The surface antigens including CD34,CD29, CD11b, CD45, and CD44 of mMSCs detected by flow cytometry. Each surface markers’ positive rate was presented.

Bone marrow from 615 mice was used to isolate mMSCs based on their plastic adherence properties. The isolated mMSCs growing in a monolayer displayed typical spindle‐shaped fibroblastoid morphology (Fig. 1A). The in vitro differentiation potential analysis showed that the isolated mMSCs could differentiate into adipocytes (Oil Red O staining, Fig. 1B) and osteoblasts (alkaline phosphatase staining, Fig. 1C) in the appropriate culture medium. mMSC surface markers were detected with flow cytometry. As shown, the percentage of mMSCs positive for CD29 and CD44 were 98.92% and 68.71%, respectively, while mMSCs were almost negative for the expression of hematopoietic markers CD34, CD11b, and CD45 (Fig. 1D).

### mMSCs educated by MFC‐CM

**Figure 2:**
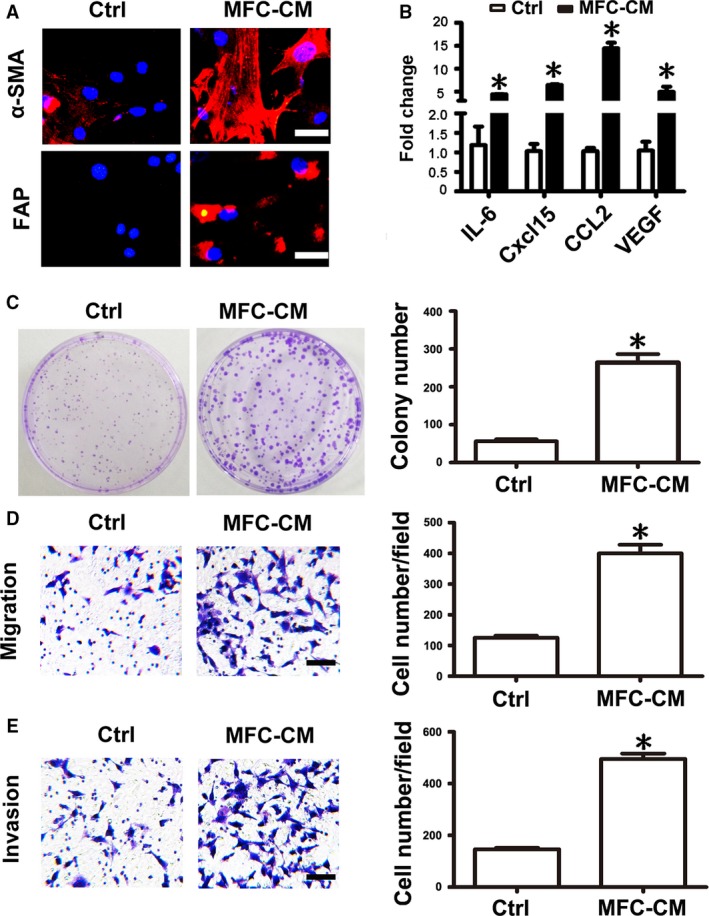
MFC‐CM induced mMSCs to acquire tumor‐promoting phenotype and function. mMSCs were attached to the plate overnight and then treated with gastric cancer cells MFC conditioned medium (MFC‐CM) for 48 h. (A) Immunofluorescence staining of *α*‐SMA and FAP in mMSCs, Magnification: ×200, Scale bar = 50 μm. (B) *q*RT‐PCR of cytokines including IL‐6, Cxcl15, CCL2, and VEGF mRNA expression in mMSCs. After treated with MFC‐CM, mMSCs were cultured in refreshed medium for 24 h and then the culture medium was collected to treat MFCs. (C) Plate cell colony formation assay. The number of colonies was counted and presented as columns. (D) Transwell migration detection. (E) Transwell invasion assay. Magnification: 100×, Scale bar = 100 μm. Representative graphs of the migrated and invasive gastric cancer cells were shown. Cell number of each field was counted and presented as columns. Data were presented as means ± SD. *P <* *0.05.

MSCs have been intensively studied as an important cellular source for tumor stromal cells. To analyze whether the murine gastric cancer cell line MFC could educate mMSCs to acquire tumor stromal cell‐like phenotype and function, we collected culture medium from MFCs and treated mMSCs for 48 h. As indicated by immunofluorescence staining and *q*RT‐PCR, after culture in MFC‐CM, mMSCs expressed higher levels of *α*‐SMA protein, FAP protein, and several tumor‐promoting cytokine genes including interleukin (IL)‐6, chemokine (C‐X‐C motif) ligand 15 (CXCL15), chemokine (C‐C motif) ligand 2 (CCL2), and vascular endothelial growth factor (VEGF) (Fig. 2A and B). To test the change in the tumor‐promoting function of mMSCs after exposure to MFC‐CM, we performed plate clone formation analysis and observed that the number and size of gastric cancer cell colonies were remarkably greater and larger in the MFC‐CM‐treated mMSC group than in the control group (Ctrl) (Fig. 2C). Furthermore, transwell migration and invasion experiments also showed that there were significantly increased numbers of migrated and invasive gastric cancer cells in the MFC‐CM‐treated mMSC group (Fig. 2D and E). These data suggest that murine gastric cancer cell line MFC conditioned medium could endow mMSCs with tumor stromal cell‐like phenotype and function.

### Knockdown of mmu‐miR‐155‐5p elicited a similar effect as MFC‐CM on mMSCs

**Figure 3:**
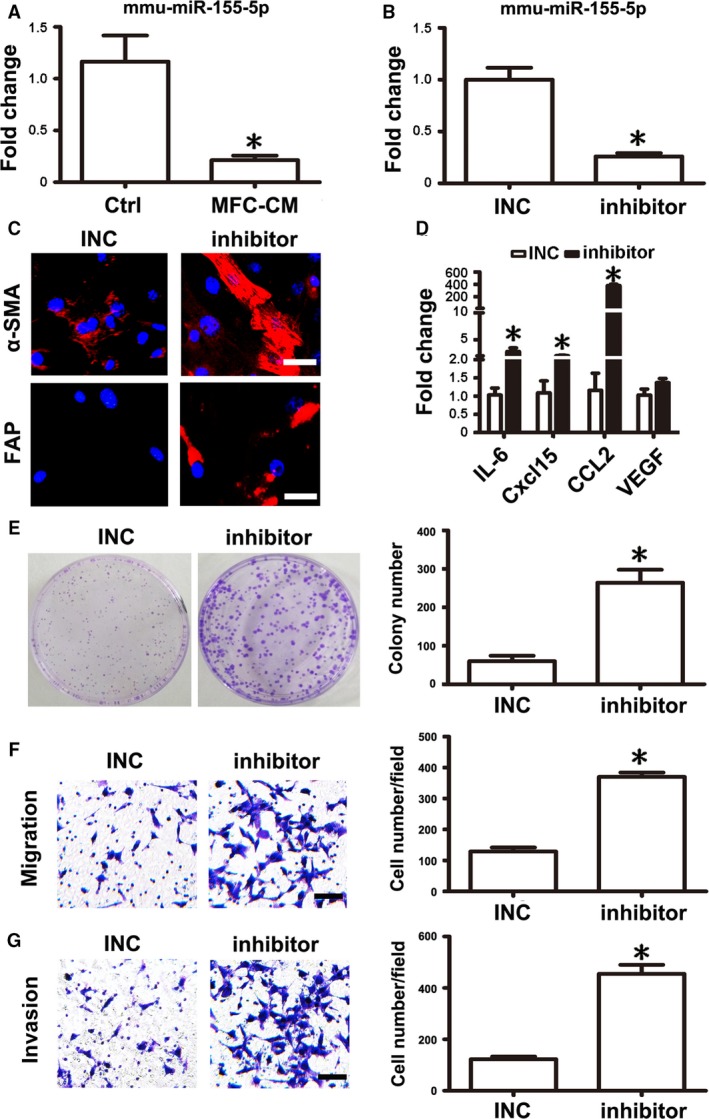
mmu‐miR‐155‐5p inhibition conferred mMSCs with tumor‐promoting phenotype and function. (A) Fold change of mmu‐miR‐155‐5p levels measured by *q*RT‐PCR in mMSCs after treated with MFC‐CM. (B) mMSCs were transfected with mmu‐miR‐155‐5p inhibitor (inhibitor) to suppress the expression of mmu‐miR‐155‐5p. Inhibitor negative control (INC) was set as a control. (C) Immunofluorescence staining of *α*‐SMA and FAP in mMSCs. (D) *q*RT‐PCR of cytokine mRNA expression levels in mMSCs. e.g., Functional studies for mMSCs on gastric cancer cells. (E, Plate cell colony formation assay. (F) Migration analysis. (G) Invasion assay. Data were presented as means ± SD. *P <* *0.05.

miRNAs have been demonstrated to be highly conserved among different species. Our previous study has revealed that knockdown of hsa‐miR‐155‐5p could induce the transition of BM‐MSCs into GC‐MSC‐like cells. According to the miRBase database (http://www.mirbase.org/), hsa‐miR‐155‐5p and mmu‐miR‐155‐5p have the same seed sequences. Therefore, we focused on mmu‐miR‐155‐5p and tested whether this miRNA was deregulated in mMSCs after educated by MFC‐CM firstly. As we expected, mmu‐miR‐155‐5p expression levels were indeed obviously reduced in mMSCs after treatment with MFC‐CM (Fig. 3A). Based on this result, we hypothesized that knockdown of mmu‐miR‐155‐5p in mMSCs may replicate the effect of MFC‐CM on mMSCs. We therefore suppressed the expression level of mmu‐miR‐155‐5p in mMSCs by miRNA inhibitor transfection. Inhibitor negative control sequence (INC) was used as the corresponding control (Fig. 3B). Immunofluorescence staining analysis showed that knockdown of mmu‐miR‐155‐5p induced *α*‐SMA and FAP expression in mMSCs (Fig. 3C). Increased mRNA levels of IL‐6, Cxcl15, and CCL2 were also detected using *q*RT‐PCR in mMSCs transfected with mmu‐miR‐155‐5p inhibitor. However, the VEGF mRNA expression level was not altered in mMSCs after transfection (Fig. 3D). Furthermore, we performed functional experiments to analyze the change in the tumor‐promoting function of mMSCs after mmu‐miR‐155‐5p inhibitor transfection. The number of cell colonies, as well as migrated and invasive gastric cancer cells, was significantly increased in mMSCs transfected with mmu‐miR‐155‐5p inhibitor (Fig. 3E–G), which was similar to those observed for mMSCs educated by MFC‐CM.

### NF‐*κ*B activity was necessary for mmu‐miR‐155‐5p inhibitor action on mMSCs

**Figure 4:**
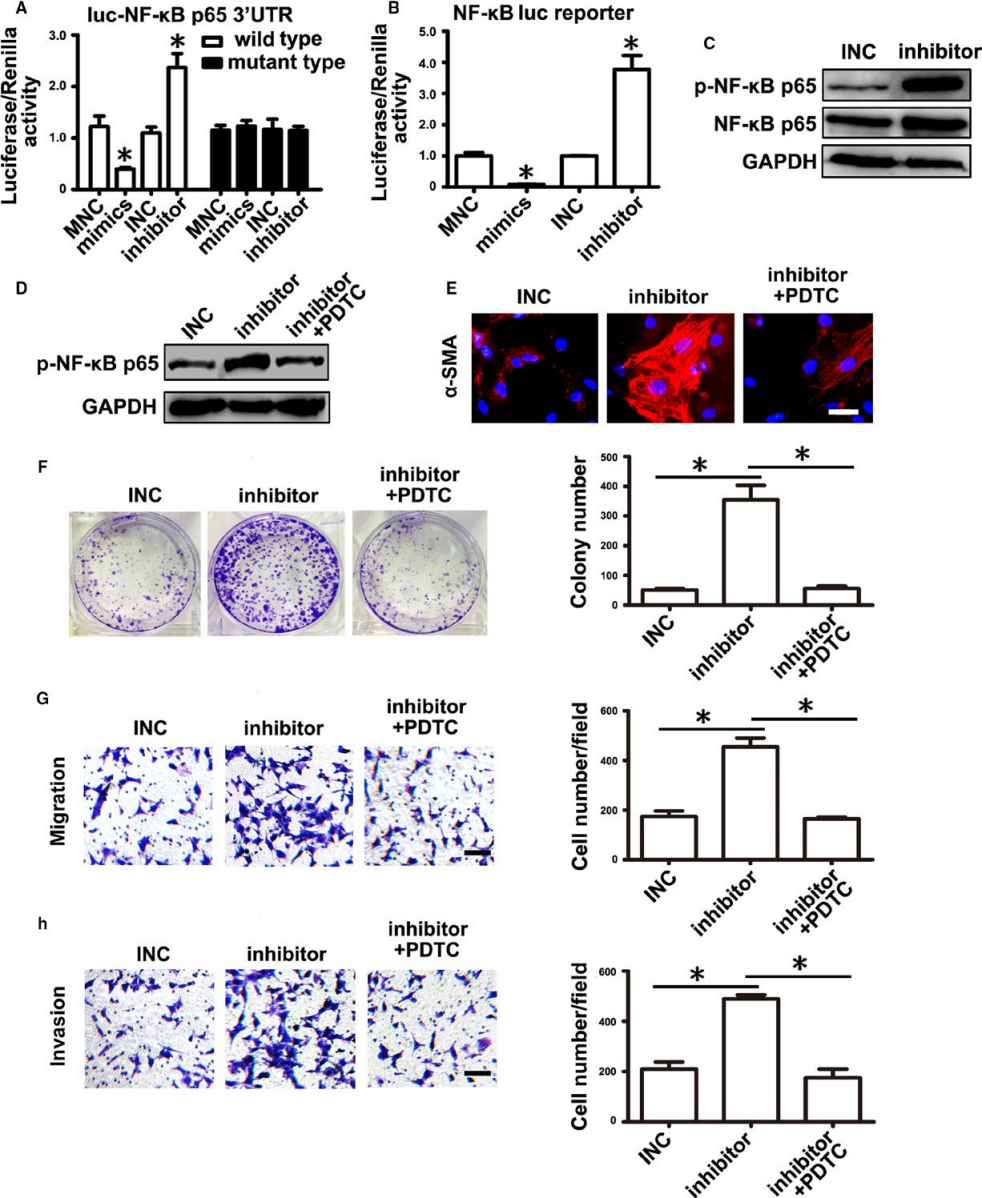
mmu‐miR‐155‐5p inhibitor regulated mMSCs depending on NF‐*κ*B activation. (A,B) Luciferase activity assay. mmu‐miR‐155‐5p mimics or inhibitor was cotransfected with luciferase reporters. Mimic negative control (MNC) or INC was set as a corresponding control. (A) luc‐NF‐*κ*B p65 3ʹ UTR pmirGLO dual‐luciferase miRNA target expression vector containing the sequences of the predicted binding sites in NF‐*κ*B p65 mRNA 3ʹ UTR or mutant the predicted binding sites. (B) NF‐*κ*B luc reporter NF‐*κ*B luciferase reporter plasmid. (C) Western blot analysis of NF‐*κ*B p65 and its phosphorylation level in mMSCs transfected with miRNA inhibitor. (D–H) mMSCs were pretreated with PDTC and then transfected with miRNA inhibitor. (D) Western blot analysis. (E) *α*‐SMA detection in mMSCs. (F–H) Functional studies for mMSCs on gastric cancer cells. (F) Cell colony formation assay. (G) Migration analysis. (H) Invasion assay. Data were presented as means ± SD. *P <* *0.05.

Based on the conserved role of miRNAs in different species and prediction information provided by the TargetScan algorithm, we constructed a luciferase reporter construct with murine NF‐*κ*B p65 mRNA 3ʹ UTR containing the predicted mmu‐miR‐155‐5p binding sites (wild‐type Luc‐NF‐*κ*B p65 3ʹ UTR), which was cotransfected with mmu‐miR‐155‐5p mimics (mimics) or mmu‐miR‐155‐5p inhibitor (inhibitor). Mimics negative control (MNC) and INC were used as the corresponding controls. Compared to the MNC group, relative luciferase activity was significantly reduced in the mimics group. In turn, relative luciferase activity was remarkably increased by inhibitor compared to INC (Fig. 4A). To evaluate the importance of the binding sites for mmu‐miR‐155‐5p regulating NF‐*κ*B p65, we changed the binding sites sequences and constructed a mutant reporter vector (mutant type Luc‐NF‐*κ*B p65 3′UTR) with which we then performed cotransfection. The relative luciferase activity was not altered whether cotransfected with mimics or inhibitor (Fig. 4A). Meanwhile, we also obtained a NF‐*κ*B luciferase reporter plasmid (NF‐*κ*B luc reporter), which was used to analyze whether mmu‐miR‐155‐5p affected NF‐*κ*B activity. The results showed that mmu‐miR‐155‐5p mimics significantly suppressed NF‐*κ*B activation, while mmu‐miR‐155‐5p inhibitor increased its activation (Fig. 4B). Moreover, western blot analyses validated that knockdown of mmu‐miR‐155‐5p significantly increased NF‐*κ*B p65 protein level and its phosphorylation level in mMSCs (Fig. 4C).

To analyze whether NF‐*κ*B activation was involved in mmu‐miR‐155‐5p inhibitor regulating mMSCs, we pretreated mMSCs with the NF‐*κ*B activity inhibitor PDTC for two hours and then transfected them with miRNA inhibitors for 48 h. Western blot analysis showed that the phosphorylated protein level of NF‐*κ*B p65 in mMSCs was evidently increased by mmu‐miR‐155‐5p inhibitor, but was reduced to the basal level by PDTC pretreatment (Fig. 4D). Consistently, enhanced expression of *α*‐SMA induced by mmu‐miR‐155‐5p inhibitor in mMSCs diminished after PDTC treatment (Fig. 4E). Functional tests showed that changes in the number of cell colonies and number of migrated and invasive gastric cancer cells that were increased in mMSCs transfected with miRNA inhibitor were reversed after PDTC pretreatment and were similar to the number detected in the INC group (Fig. 4F–H).

### mmu‐miR‐155‐5p overexpression partially abolished the effect of MFC‐CM on mMSCs

**Figure 5:**
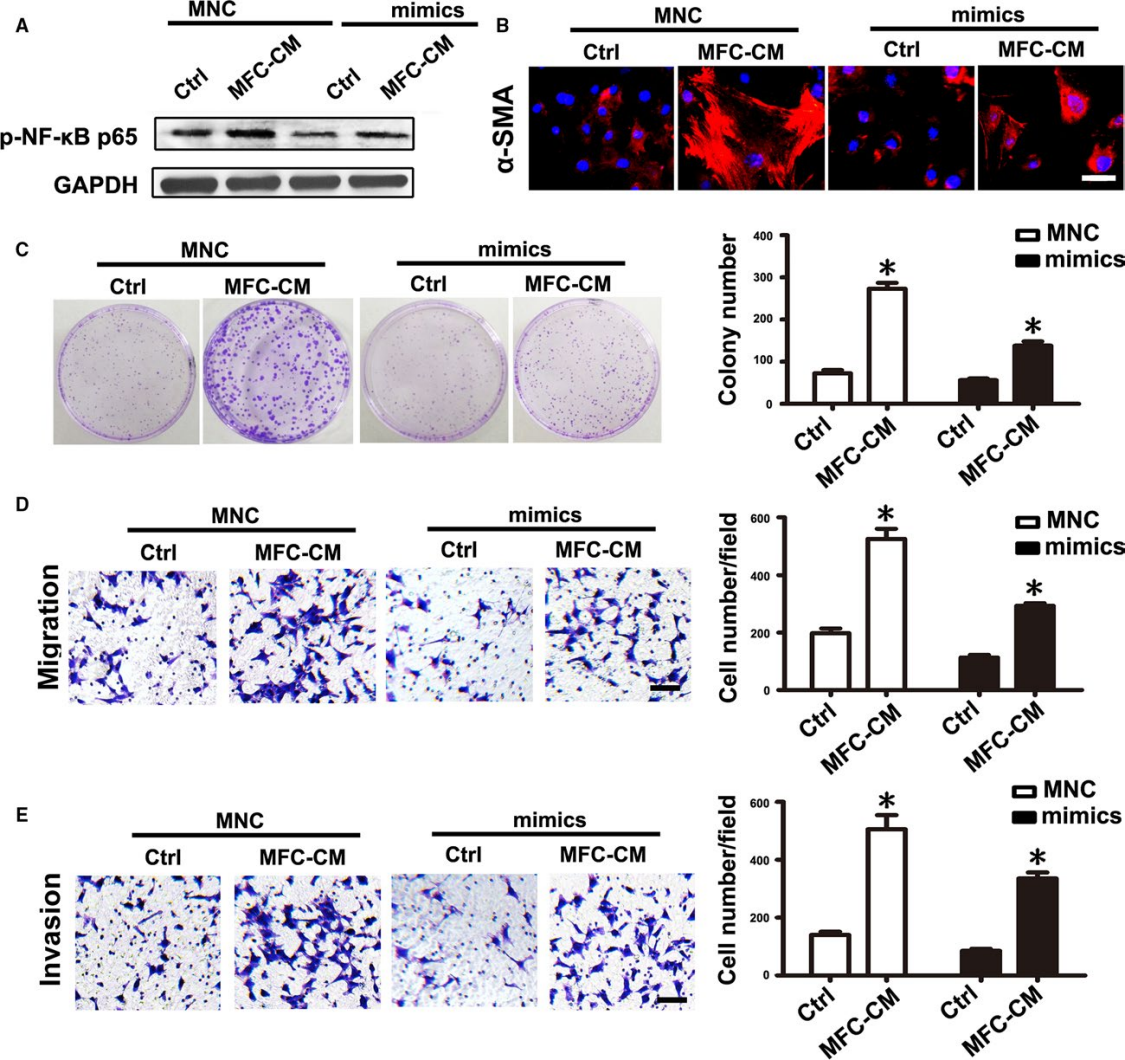
mmu‐miR‐155‐5p mimics partially eliminated the effect of MFC‐CM on mMSCs. mMSCs were transfected with mmu‐miR‐155‐5p mimics before treated with MFC‐CM. (A) Western blot analysis of the phosphorylation levels of NF‐*κ*B p65 in mMSCs. (B) *α*‐SMA detection in mMSCs. (C–E) Functional studies for mMSCs on gastric cancer cells. (C) Cell colony formation assay. (D) Migration assay. (E) Invasion analysis. Data were presented as means ± SD. *P <* *0.05.

As knockdown of miR‐155‐5p could reproduce the effect of MFC‐CM on mMSCs, we asked if miR‐155‐5p overexpression could also abolish the role of MFC‐CM in educating mMSCs. mMSCs were transfected with miRNA mimics to overexpress mmu‐miR‐155‐5p before treatment with MFC‐CM. Western blot analysis showed that in both the MNC and mimics group, the phosphorylated levels of NF‐*κ*B p65 were obviously increased in mMSCs after MFC‐CM treatment. mmu‐miR‐155‐5p overexpression slightly weakened the effect of MFC‐CM on NF‐*κ*B p65 activation (Fig. 5A). The immunofluorescence intensity of *α*‐SMA induced by MFC‐CM in mMSCs was partially suppressed by mmu‐miR‐155‐5p mimics (Fig. 5B). The number and size of gastric cancer cell colonies in the MFC‐CM groups were greater and larger than those in the corresponding control groups. Similarly, mmu‐miR‐155‐5p mimics only slightly reduced the number of cell colonies and made the colony size smaller (Fig. 5C). In the MNC group, the number of migrated and invasive gastric cancer cells was clearly increased in the MFC‐CM group. Nonetheless, while mmu‐miR‐155‐5p overexpression reduced the corresponding number of cells in the MFC‐CM group, the number was still greater than that in the control group (Fig. 5D and E). These data together indicate that mmu‐miR‐155‐5p overexpression partially abolished the effect of MFC‐CM on mMSCs.

### mMSCs education by MFC‐CM was dependent on NF‐*κ*B activity

**Figure 6:**
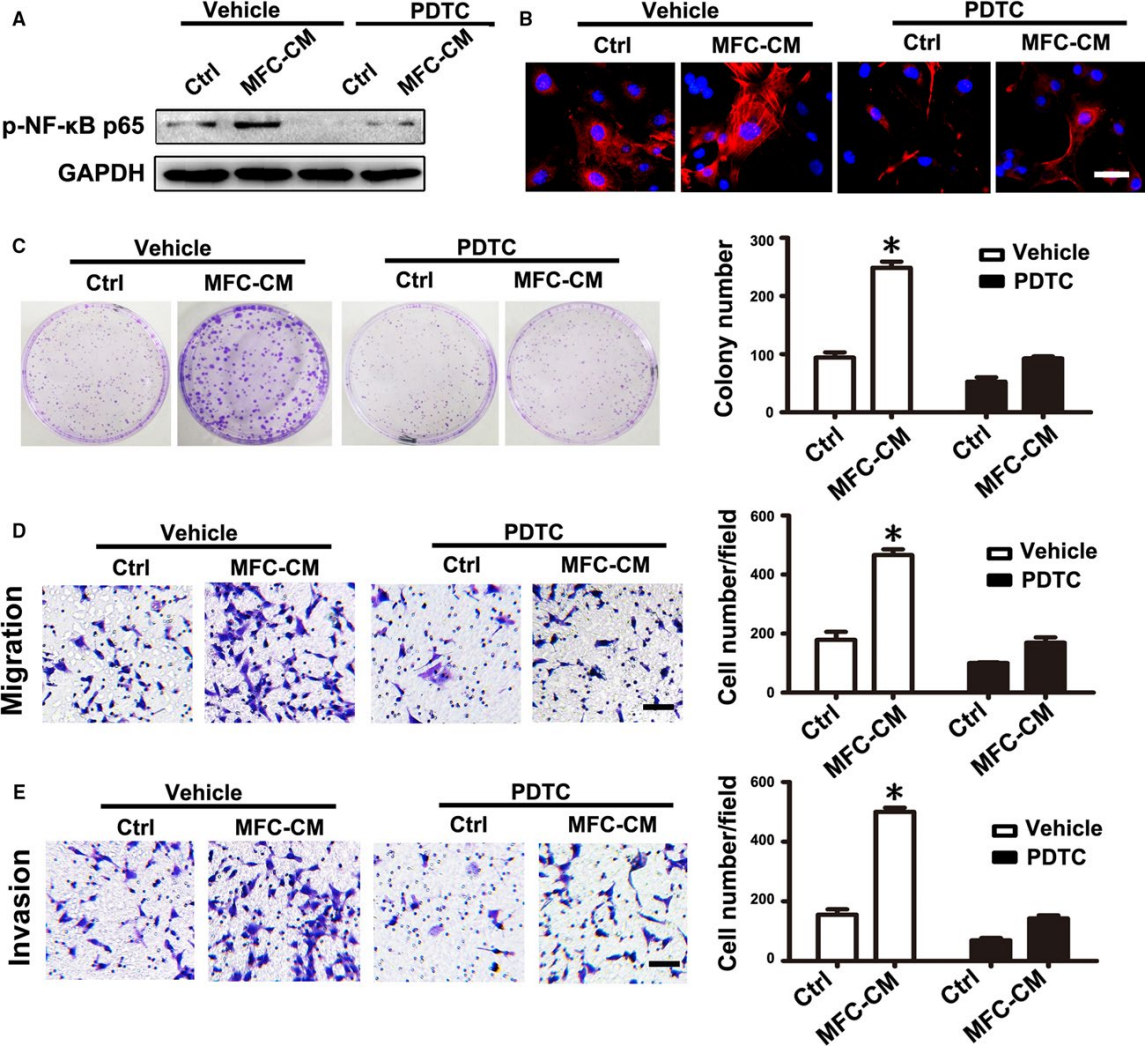
PDTC pretreatment abolished the effect of MFC‐CM on mMSCs. mMSCs were pretreated with PDTC before culture in MFC‐CM. (A) Western blot analysis of the phosphorylation levels of NF‐*κ*B p65 in mMSCs. (B) Immunofluorescence staining of *α*‐SMA in mMSCs. (C–E) Functional studies for mMSCs on gastric cancer cells. (C) Cell colony formation assay. (D) Migration assay. (E) Invasion analysis. Data were presented as means ± SD.*P <* *0.05.

To further evaluate whether NF‐*κ*B activity was necessary for the education of mMSCs by MFC‐CM, we used PDTC to inhibit NF‐*κ*B activity in mMSCs before treatment with MFC‐CM. The phosphorylated levels of NF‐*κ*B p65 detected by western blot showed that PDTC pretreatment obviously reduced the phosphorylation of NF‐*κ*B p65 in mMSCs induced by MFC‐CM (Fig. 6A). Immunofluorescence staining analysis showed that PDTC pretreatment resulted in nearly undetectable levels of *α*‐SMA expression in mMSCs treated with MFC‐CM (Fig. 6B). In functional studies, the increase in the number of cell colonies, as well as migrated and invasive gastric cancer cells in the MFC‐CM group, was significantly reduced by PDTC pretreatment and was near to the numbers detected in the control group without PDTC treatment (Fig. 6C–E). These results suggest that NF‐*κ*B activity is necessary for MFC‐CM inducing mMSCs to acquire tumor‐promoting phenotype and function.

## Discussion

It is well documented that the reactive tumor stroma plays a pivotal role in tumor progression. Among the diverse tumor stromal cells, CAFs have been identified as essential components contributing to the fate of tumor cells 28, 29. Based on the innate tropism of BM‐MSCs to inflammatory sites and their multipotency, accumulating evidence has shown that BM‐MSCs are an important cellular source of tumor stromal cells, particularly CAFs. Although molecular markers defining CAFs are not specific, it is generally accepted that *α*‐SMA, FAP, and several tumor‐promoting inflammatory cytokines characterize the CAF phenotype 12, 30. Based on alterations in phenotype and tumor‐promoting functions, increasing numbers of studies have demonstrated that MSCs can be converted into CAF‐like cells under exposure to tumor cell‐conditioned medium in vitro, indicating the essential role of soluble factors secreted by tumor cells for this process. Several factors such as transforming growth factor (TGF)‐*β*1 10, 14, osteopontin 15, 78 kDa glucose‐regulated protein (GRP78) 13, and basic fibroblast growth factor (bFGF) 31 were highly enriched in tumor cell‐conditioned medium and were required for inducing CAF‐like differentiation of BM‐MSCs. Furthermore, a recent study revealed that tumor cell‐derived exosomes endowed BM‐MSCs with a tumor‐favorable phenotype and tumor‐promoting capability 20, 21. This information suggests that tumor cell‐conditioned medium contains important signals for the education of BM‐MSCs. Thus, in the present study, we first considered using conditioned medium (CM) from gastric cancer cell line MFC to establish an in vitro education model of mMSCs.

Compared to BM‐MSCs, cancer tissue‐derived MSCs have similar characteristics, but exhibit prominent tumor‐promoting phenotype and function 26. Cancer tissue‐derived MSCs have the same potential for adipogenic and osteogenic differentiation as BM‐MSCs, which indicates that BM‐MSCs educated by tumor cell‐CM are likely converted into cancer tissue‐derived MSC‐like cells. It must be noted, however, that fibroblasts and MSCs share many similarities. A study has shown that CAFs also have the capacity to differentiate into osteocytes, chondrocytes, and adipocytes 32, which makes it difficult to distinguish fibroblasts from MSCs 33. CAFs from MSCs may thus be a subset of “specialized” MSCs 32, suggesting that cancer cells induce transition of BM‐MSCs into another kind of cells which could be generally described as cancer‐associated MSCs. In our study, mouse gastric cancer cell‐CM treatment resulted in higher expression levels of *α*‐SMA, FAP, and inflammatory cytokines and the acquisition of tumor‐promoting capability in mMSCs, which indicates that we had successfully established the education model using MFC‐CM to induce the transition of mMSCs into cancer‐associated MSCs.

It is well known that resident normal fibroblasts (NFs) are another important source of CAFs. Recently, emerging reports have described how miRNAs were frequently deregulated in CAFs and that modifying the expression of these miRNAs could reprogram NFs into CAFs 23, 24, 25. Previously, miRNA array analysis showed that there are many miRNAs deregulated in GC‐MSCs 34. We hypothesized that miRNAs deregulated in GC‐MSCs may participate in reprogramming BM‐MSCs into GC‐MSCs. Our previous work validated that miR‐155‐5p expression was downregulated in GC‐MSCs. To mimic the suppressive state of miR‐155‐5p expression in GC‐MSCs, we used miRNA inhibitor transfection to directly reprogram BM‐MSCs into gastric cancer tissues‐derived MSC‐like cells via NF‐*κ*B activation 26. Consistently, in the present study, we also found that mmu‐miR‐155‐5p expression was significantly suppressed in mMSCs after education by MFC‐CM. mmu‐miR‐155‐5p inhibitor transfection directly induced transition of mMSCs into cancer‐associated MSCs via NF‐*κ*B activation. These data together emphasize that the function of miR‐155‐5p is conserved, which may be attributed to the same seed sequences in miR‐155‐5p shared by human and mouse species.

VEGF is important for the promotion of cancer progression by tumor stromal cells and has been shown to be highly expressed in CAFs and cancer tissue‐derived MSCs 24, 26, 35. VEGF mRNA levels were significantly increased in mMSCs after education by gastric cancer cell‐CM. Unexpectedly, while mmu‐miR‐155‐5p knockdown also endowed mMSCs with tumor‐promoting phenotype and function, VEGF expression was not affected in mMSCs after mmu‐miR‐155‐5p inhibitor transfection. To further evaluate the role of miR‐155‐5p in mediating gastric cancer cell‐CM induced transition of mMSCs into cancer‐associated MSCs, we observed that mmu‐miR‐155‐5p overexpression could not completely but only partially abolished the effect of gastric cancer cell‐CM on mMSCs. These data together indicate that miR‐155‐5p is not the sole miRNA mediating the education of mMSCs by MFC‐CM. Moreover, several previous studies have demonstrated that at least three deregulated miRNAs together could reprogram NFs into CAFs 23, 24, which further suggests that other important miRNAs mediating the education of BM‐MSCs by gastric cancer cell‐CM need to be identified in the future.

NF‐*κ*B p65 was validated as a target of mmu‐miR‐155‐5p, and its phosphorylation levels were negatively regulated by mmu‐miR‐155‐5p. Previously, we showed that miR‐155‐5p inhibitor activated NF‐*κ*B p65 by upregulating inhibitor of nuclear factor kappa‐B kinase subunit epsilon (IKBKE) 26. Based on the conserved sequences of the miRNAs, we think that mmu‐miR‐155‐5p may regulate NF‐*κ*B p65 activation in the same manner. The NF‐*κ*B signaling pathway is tightly associated with a proinflammatory phenotype of MSCs induced by tumor cells 26, 36. To further analyze the role of the NF‐*κ*B pathway in the education of mMSCs by gastric cancer cell‐CM, we used the NF‐*κ*B inhibitor PDTC to suppress the NF‐*κ*B pathway in mMSCs before treatment with MFC‐CM. We found that NF‐*κ*B pathway inhibition significantly blocked the effect of MFC‐CM on mMSCs, which suggest that the NF‐*κ*B pathway may be the key signaling involved in the education process. miR‐155‐5p overexpression was unable to completely suppress NF‐*κ*B p65 activation induced by gastric cancer cell‐CM, which at least provides an explanation for why miR‐155‐5p could not fully abolish the effect of MFC‐CM on mMSCs. As is well known, one miRNA may regulate many different targets, while one mRNA can be targeted by many different miRNAs. The foregoing information implies that other miRNAs targeting the NF‐*κ*B pathway might be involved in the education process. Target regulation of the NF‐*κ*B pathway may thus serve as a criterion for screening other important miRNAs which may potentially serve as therapeutic targets to modulate the tumor microenvironment.

In conclusion, miR‐155‐5p may not be the sole miRNA mediating the gastric cancer cell‐CM‐induced transition of BM‐MSCs into cancer‐associated MSCs, but the NF‐*κ*B signaling pathway is necessary for this process. Our findings provide a deeper insight into the role of miR‐155‐5p in the transition process and indicate new directions for exploring additional miRNAs involved in the underlying mechanism of cell education.

## Conflict of Interest

All authors declare that they have no conflict of interest.

## Supporting information


**Table S1**. Cytokines mRNAs primers sequences for *q*RT‐PCR and the conditions of amplification.

**Table S2**. Sequences of the synthesized oligonucleotides.
