# Correction to “The role of mmu‐miR‐155‐5p‐NF‐κB signaling in the education of bone marrow‐derived mesenchymal stem cells by gastric cancer cells”Wang, M., Yang, F., Qiu, R., Zhu, M., Zhang, H., Xu, W., Shen, B. and Zhu, W. (2018), The role of mmu‐miR‐155‐5p‐NF‐*κ*B signaling in the education of bone marrow‐derived mesenchymal stem cells by gastric cancer cells. Cancer Med, 7: 856–868. 10.1002/cam4.1355

**DOI:** 10.1002/cam4.7469

**Published:** 2024-07-08

Figure 5E was incorrect in the original version. It has been updated as shown below:

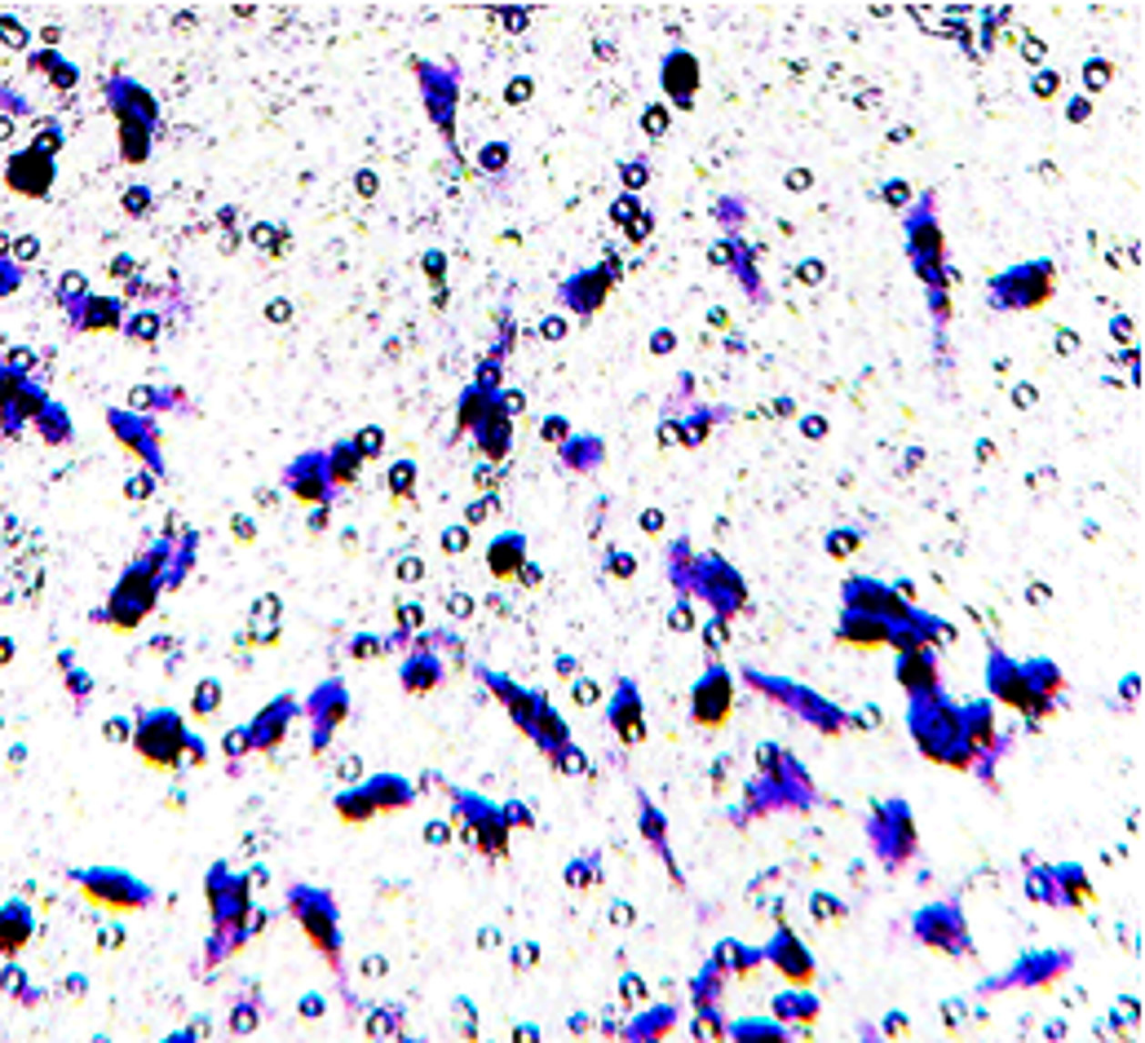

We apologize for this error.

