# Supplementary material for: The role of mmu‐miR‐155‐5p‐NF‐κB signaling in the education of bone marrow‐derived mesenchymal stem cells by gastric cancer cells
Source: Cancer Med. 2018 Feb 14;7(3):856–68. doi: 10.1002/cam4.1355 (PMC5852371; doi:10.1002/cam4.1355)
Supplement: Supplementary file 1 — Table S1. Cytokines mRNAs primers sequences for qRT‐PCR and the conditions of amplification. [file CAM4-7-856-s001.doc]

**Supplementary Table 1**

**Cytokines mRNAs primers sequences for *q*RT-PCR and the conditions of amplification**

| **Genes** | **Primers sequences (5’-3’)** | **Annealing**  **Temperatures(℃)** | **Product**  **length(bp)** |
| --- | --- | --- | --- |
| mβ-actin | For†:GACCTGTACGCCAACACAGT | 58 | 129 |
|  | Rev‡:CTCAGGAGGAGCAATGATCT |  |  |
| mIL-6 | For†::AAGTCCGGAGAGGAGACTTC  Rev‡:TGGATGGTCTTGGTCCTTAG | 63 | 257 |
| mCxcl15 | For†::GGCTGTCCTTAACCTAGGCATCT  Rev‡:GGTCCTCAGGTAGGAACCTGTTAGT | 58 | 487 |
| mCCL2 | For†::GTCCCTGTCATGCTTCTGG  Rev‡:GTGCTTGAGGTGGTTGTGG | 56 | 407 |
| mVEGF | For†::ACGGGAGACAATGGGATGA  Rev‡:AGTGGAGGAGCGAGCTGAA | 58 | 333 |

† For, forward primer; ‡ Rev, reverse primer. m, mouse.
