# Supplementary material for: The role of mmu‐miR‐155‐5p‐NF‐κB signaling in the education of bone marrow‐derived mesenchymal stem cells by gastric cancer cells
Source: Cancer Med. 2018 Feb 14;7(3):856–68. doi: 10.1002/cam4.1355 (PMC5852371; doi:10.1002/cam4.1355)
Supplement: Supplementary file 2 — Table S2. Sequences of the synthesized oligonucleotides. [file CAM4-7-856-s002.doc]

**Supplementary Table 2**

**Sequences of the synthesized oligonucleotides**

| **Oligonucleotides name** | **Sequences** | |
| --- | --- | --- |
|  | **Sense (5’-3’)** | **Antisense (5’-3’)** |
| mimics negative control (MNC) | UUCUCCGAACGUGUCACGUTT | ACGUGACACGUUCGGAGAATT |
| mmu-miR-155-5p mimics  (mimics) | UUAAUGCUAAUUGUGAUAGGGGU | CCCUAUCACGAUUAGCAUUAAUU |
| inhibitor negative control (INC) | CAGUACUUUUGUGUAGUACAA | |
| mmu-miR-155-5p inhibitor  (inhibitor) | ACCCCUAUCACAAUUAGCAUUAA | |
| NF-κB p65 mRNA 3’UTR containing the binding sites  (wild type) | CTAGCGGCCGCTAGTTGCTTTCGCAGG**AGCATTAA**CCTCCTGGAGG | TCGACCTCCAGGAGG**TTAATGCT** CCTGCGAAAGCAACTAGCGGCCGCTAGAGCT |
| NF-κB p65 mRNA 3’UTR containing the mutant sites  (mutant type) | CTAGCGGCCGCTAGTTGCTTTCGCAGG*G****TACGGC***ACCTCCTGGAGG | TCGACCTCCAGGAGGT***GCCGTAC*** CCTGCGAAAGCAACTAGCGGCCGCTAGAGCT |

Sequences in bold indicate the predicted binding sites;

Sequences in bold and italic indicate the mutant sites.
